# Inter-fraction heart displacement during voluntary deep inspiration breath hold radiation therapy without visual feedback measured by daily CBCT

**DOI:** 10.3389/fonc.2022.936088

**Published:** 2022-08-18

**Authors:** Sofian Benkhaled, Carolina Gomes da Silveira Cauduro, Nicolas Jullian, Antoine Desmet, Diana Rodriguez, Younes Jourani, Dirk Van Gestel, Alex De Caluwé

**Affiliations:** ^1^ Department of Radiation-Oncology, Institut Jules Bordet-Université Libre de Bruxelles, Brussels, Belgium; ^2^ Department of Medical-Physics, Institut Jules Bordet-Université Libre de Bruxelles, Brussels, Belgium

**Keywords:** breast cancer, deep inspiration breath-hold, IGRT, cone-beam-computed-tomography, radiation therapy

## Abstract

**Purpose/Objective:**

Deep Inspiration Breath Hold (DIBH) is now considered as the standard of care for many breast cancer patients. However, there are still uncertainties about the dose given to the heart, and it is unknown if patients may improve voluntary DIBH depth by gaining experience during treatment. In this study, we will examine the interfractional three-dimensional (3D) heart displacement throughout voluntary DIBH (vDIBH) radiotherapy by means of daily cone-beam computed tomography (CBCT).

**Material and methods:**

Two hundred twenty-five unique CBCTs from 15 patients treated in 15 fractions were analyzed. During CBCT, a vDIBH was conducted without any visual feedback. Patients performed their DIBH freely after receiving explanations and training. After daily CBCT matching to the chest wall (CW), surface-guided radiation therapy (SGRT) tracked DIBH depth to ensure that the CW position was the same as the daily acquired CBCT. The CBCTs were retrospectively registered to the DIBH planning-CT to calculate daily changes in heart displacement relative to the CW.

**Results:**

The mean displacement of the heart during DIBH treatment relative to the DIBH planning-CT was as follows: 1.1 mm to the right, interquartile range (IQR) 8.0; 0.5 mm superiorly, IQR 4.8; and 0 mm posteriorly, IQR 6.4. The Spearman correlation coefficients (r_s_) were -0.15 (p=0.025), 0.04 (p=0.549), and 0.03 (p=0.612) for the X, Y, and Z directions, respectively. The differences in median heart displacement were significant: Friedmann rank sum test p=0.031 and pairwise comparison using the Wilcoxon rank-sum test were p=0.008 for X and Y; p=0.33 for X and Z; and p=0.07 for Y and Z. The total median heart motion was δ_tot median_= 7.26 mm, IQR= 6.86 mm.

**Conclusion:**

During DIBH, clinicians must be aware of the wide range of intra- and inter-individual heart position variations. The inter-individual heterogeneity shown in our study should be investigated further in order to avoid unexpected cardiac overexposure and to develop a more accurate heart dose-volume model.

## Introduction

External beam radiotherapy (RT) after breast-conservative surgery reduces the risk of recurrence and breast cancer mortality (1). RT and other systemic cancer therapy have improved most early breast cancer patients’ prognoses, allowing them to become long-term cancer survivors (2). Therefore, prevention and management of treatment-related toxicities are crucial (2). Compared to breast cancer-free women, patients with a history of breast cancer are more likely to die from cardiovascular disease (3, 4). Post-mastectomy RT trials published in the 1960s–1970s revealed an excess of mortality due to heart disease and lung cancer (1). Darby et al. showed that the rates of major coronary events (MCEs) increased linearly with the mean heart dose (MHD), with an estimated risk of 7.4% per gray (Gy) without threshold (5). These results were based on trials using outdated two-dimensional RT, leading to high heart dose and cardiovascular toxicity (5). Van den Bogaard et al. validated the Darby et al. model and showed that MHD alone was a poor predictor of MCE compared to the volume of the left ventricle receiving 5 Gy (LV-V5) (6). However, due to its anatomical location and the RT techniques employed (3D conformal), the LV received the highest dose compared to both atriums and the right ventricle, which may represent a bias (6). The Early-Heart study also found that the LV-V5 parameter may be the best predictor of the development of subclinical LV dysfunction (7). Once more, this finding should be interpreted with caution since patients were mostly treated with 3D-RT (60%), and Deep Inspiration Breath-hold (DIBH) techniques were applied in only 55% of the left-side breast cancer patients (7).

In this context, high-precision RT based on CT imaging has been developed (e.g., intensity modulated radiation therapy [IMRT] and volumetric modulated radiation therapy [VMAT]) (4, 8). These technologies allow for the correct localization of target volumes, organs at risk (OARs), and beam positioning (8). As a result, RT became more conformal and accurate, however, possibly leading to geometric uncertainties between planning and delivered doses (8). The dose distribution could, in fact, vary as a result of interfraction motion (9). Indeed, Tan et al. reported that the LV and coronary arteries are the most mobile heart structures throughout the normal cardiac cycle, with displacements ranging from 3 to 8 mm (10). Furthermore, depending on the dose received by different heart structures, many pathways could contribute to heart toxicities. In order to detect patient positioning variations and organ motion, image-guided radiation therapy (IGRT), including cone-beam computed tomography (CBCT), has been developed (11). Moreover, techniques such as DIBH have been proven to decrease heart and lung doses, as with each inspiration, the lung volume increases, and the heart moves away from the chest wall (CW) (12). Compared to free-breathing, DIBH could decrease the MHD and left anterior descending artery (LAD) dose to 25%–67% and 20%–73%, respectively (12, 13).

During voluntary DIBH (vDIBH), patient positioning and real-time breathing monitoring can be obtained with tattoo skin marks, IGRT, a device placed on the patient’s CW, and/or an optical surface tracking system (14). Surface-guided radiation treatment (SGRT) was found to be non-inferior to the traditional laser-based setup in breast patients and improves patient positioning monitoring (15).

Since DIBH is an established treatment for many breast cancer patients, uncertainties about the dose received by the heart are a clinically relevant concern (12). Furthermore, vDIBH is not intuitive and must be done more than once per fraction, requiring motivation and coordination skills (9, 16). According to the UK HeartSpare study, vDIBH is similar to controlled DIBH (active breathing coordinator) in terms of reproducibility and organ sparing, while being less time-consuming (simulation and daily setup) and more appreciated by the patients and therapists (14). Prior to radiation, it is impossible to predict any common respiratory patterns for a specific patient (9). It is unknown whether patients can enhance DIBH depth by acquiring experience during treatment. A possible change might be an improvement of the vDIBH, with the heart moving further away from the CW as RT progresses because the patient gains experience and confidence. On the other hand, vDIBH depth could also deteriorate due to fatigue or lack of patient cooperation or motivation.

In this study, we aim to quantify the interfractional heart displacement (3D) of vDIBH during breast RT as measured on daily CBCT, as we hypothesize that patients might perform breath-hold differently during their treatment. Importantly, to achieve this goal and to measure the natural evolution of vDIBH during the course of treatment, patients were free to perform vDIBH during the daily CBCT to a depth they felt comfortable without receiving any visual feedback. To the best of our knowledge, this study is one of the first to look at the 3D movement of the heart using daily CBCT acquired throughout vDIBH breast radiotherapy. Such dynamic and homogenous information may be crucial for accurately estimating and understanding RT-related cardiac toxicity.

## Materials and methods

### Patients and design

Fifteen consecutive patients who required adjuvant DIBH RT to the whole left breast or CW and nodal irradiation were retrospectively analyzed. Patients received explanations on how to perform a DIBH before the simulation. In addition, patients were asked to practice at home before the simulation was done. The goal of self-practice was to increase the DIBH efficiency, endurance, and compliance to maintain 20–40 seconds (s) of consistent DIBH.

### Treatment planning

All patients were simulated and treated in a supine position with arms above the head using an adjustable arm and knee support. Four skin tattoos were drawn according to the fixed laser’s intersection on the body. Patients were instructed to perform a voluntary DIBH during CT simulation (Aquilion™ Large Bore, 3-mm slice thickness). Real-time position management (RPM) from Varian Medical Systems (Palo Alto CA, USA) was used to monitor vDIBH consistency during the scan. Targets were delineated on the vDIBH CT scan according to the ESTRO guidelines (17). Heart delineation included the pulmonary artery bifurcation to the apex of the ventricle according to the RTOG breast cancer atlas for radiation therapy planning (Radiation Therapy Oncology Group). The prescribed dose was 40.05 Gy in 15 fractions over 3 weeks. Treatment plans were calculated with Monaco 5.0 (Elekta AB, Stockholm, Sweden), and the dose was computed using a Monte Carlo algorithm. All patients were treated with VMAT (Elekta Infinity™ equipped with an Agility™ head), with a single isocenter and a single 270° arc 6 MV coplanar flattened photon beam (max 600 MonitorUnits/min). Constraints were based on the DBCG HYPO trial protocol (18).

All the plans were evaluated using global gamma assessment with 3%/3 mm criteria above a 20% maximum dosage threshold for 95% of measured points using the Delta4+ phantom. DIBH instructions and the interval between vDIBH instructions were identical for both planning and treatment.

### Treatment delivery and statistical analysis

CBCT under vDIBH condition without SGRT was acquired, with a full rotation requiring less than a minute. Patients were free to perform the vDIBH as they wished without guidance from radiation therapy technologists (RTTs). During vDIBH, a new reference surface scan was taken, which would subsequently be used during the RT session as the day’s threshold to ensure the patients performed the vDIBH in the same position as the CBCT (5-mm tolerance in all directions). Initial automatching was performed based on bone structures (clipbox to thoracic wall and/or vertebrae) and breast soft tissue. Then couch corrections were performed to register the vDIBH CBCT to the initial vDIBH planning-CT. Importantly, the chosen SGRT reference surface for the treatment was not based on the simulation CT but on the surface acquired during the vDIBH performed during CBCT. During the treatment, if vDIBH was outside the tolerance (5 mm), the beam was turned off and the RTT asked the patient to perform another vDIBH.

Daily CBCT scans were acquired before each fraction (n=15) and retrospectively 3-dimensionally rigidly registered by the same radiation oncologist to the vDIBH planning-CT. The heart position on the vDIBH planning-CT was defined as the reference position. Two different offline registrations were performed: 1) on the thoracic wall (X_bones_, Y_bones_, Z_bones_) and 2) on the heart (X_heart_, Y_heart_, Z_heart_) to calculate heart displacement relative to the thoracic wall.

The total 3D heart motion per fraction was calculated as follows: [δ_x_= (√[X_bones_-X_heart_]^2^ + [Y_bones_-Y_heart_] ^2^ + [Z_bones_-Z_heart_] ^2^)]. Descriptive statistics median and interquartile range (IQR) were calculated for the displacement of the entire heart structure delineation in each direction: X[right-left]; Y[inferior-superior]; Z[posterior-anterior] directions. The heart position on the DIBH planning-CT and the heart position on CBCT during treatment were correlated using linear regression, Wilcoxon signed-rank test, and the Friedman test. R_s_ and p-values were calculated using the Spearman correlation test. Statistical analysis was performed using R version 4.1.0, with a p*-*value <0.05 considered as significant.

## Results

Two hundred twenty-five CBCTs from 15 patients were individually analyzed. The median (Q1–Q3) age was 45.4 (32–63) years, and BMI was 29.2 kg/m³ (27.2–35.6). The median clinical target volume (CTV) and planning target volume (PTV) were 3024 cm³ (2055–5631) and 4809 cm³ (2924–8284), respectively. Concerning the OARs, the heart volume was 6652 cm³ (5355–7899), LAD 62 cm³ (27.5–92), and lungs 42363 cm³ (36003–46426).

**Figure 1:**
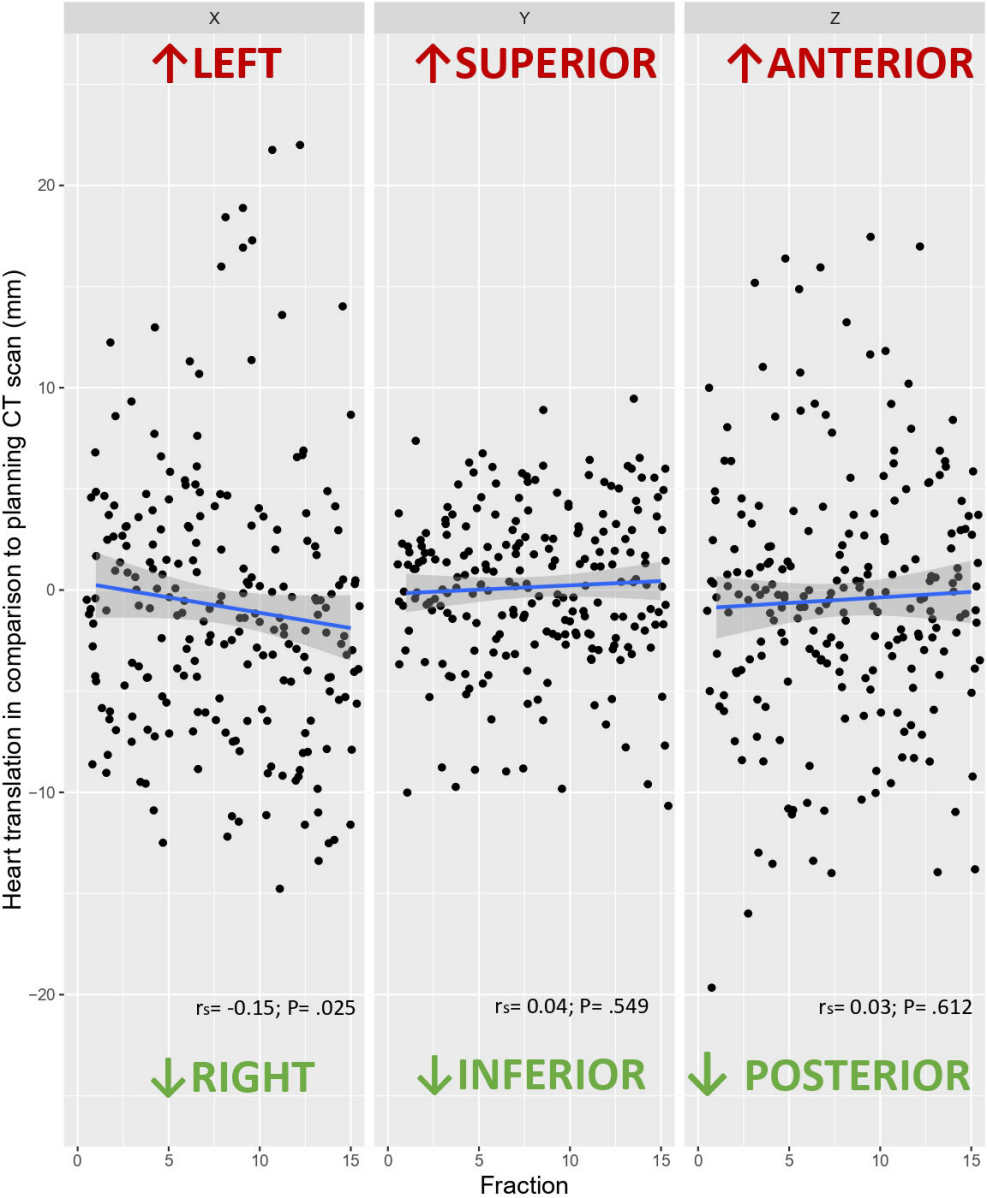
Heart translation in each axis (X, Y, Z) in comparison to the planning CT scan according to the 225 CBCTs, with the linear correlation (blue line) and its 95% confidence interval (gray area).

**Figure 2:**
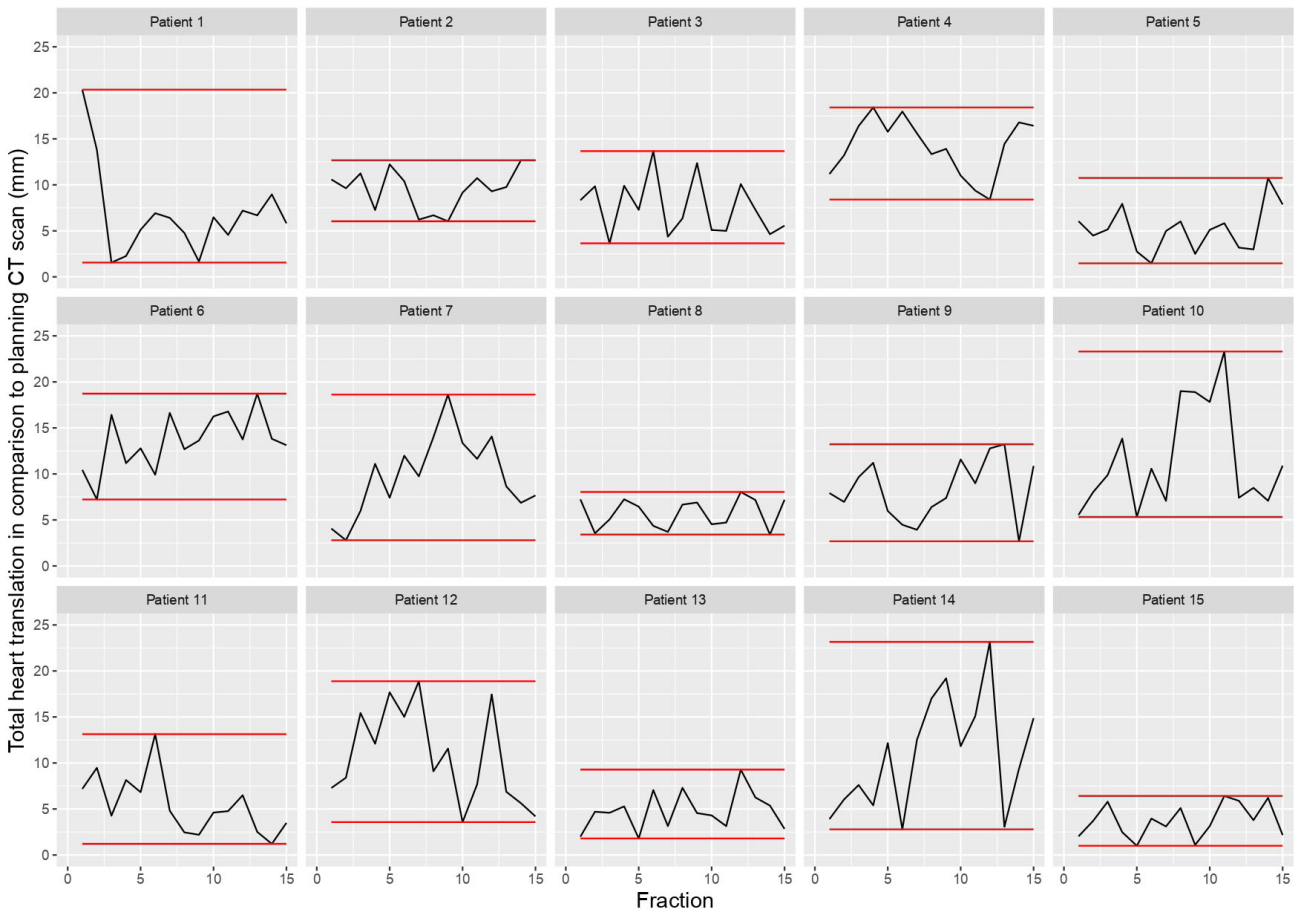
Individual 3D heart translation per patient (n = 15) in comparison to the planning CT scan, with the minimal and maximal translation (red line).

**Figure 3:**
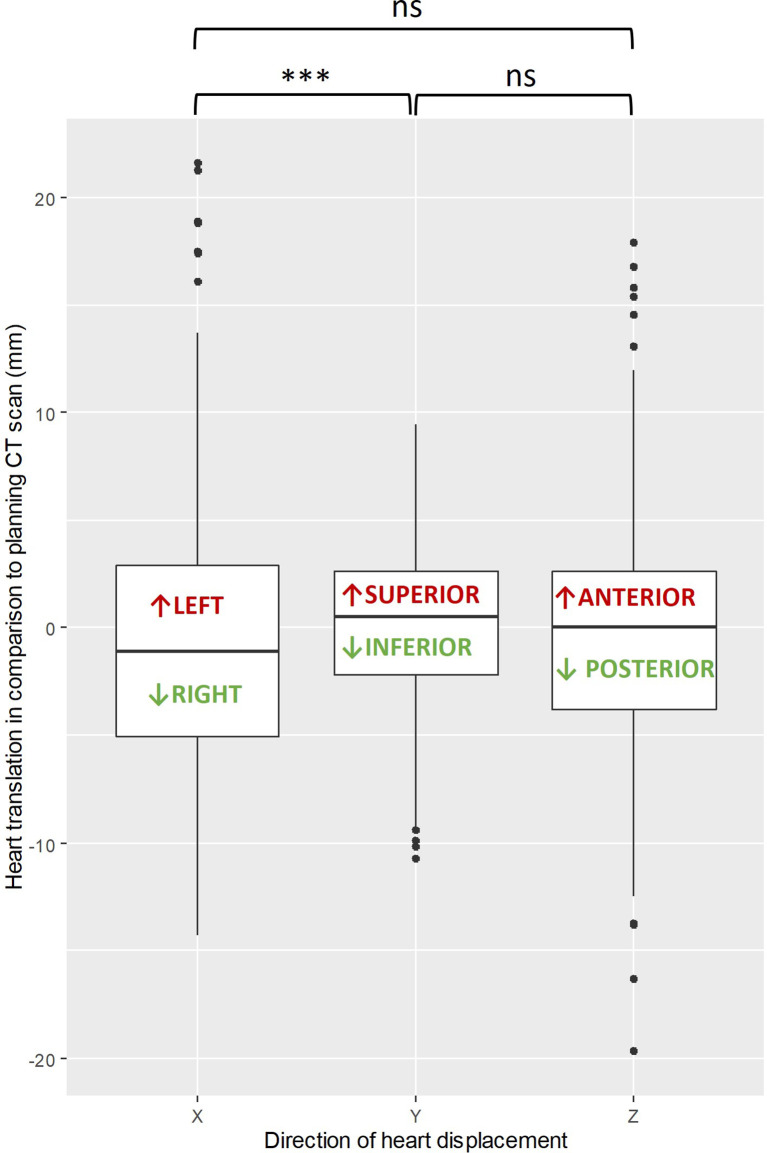
Box plot showing the differences in median heart translation (mm) in each axis (X, Y, Z), with the Wilcoxon rank-sum test comparison (black bracket). ***: p-value is less than 0.001, ns: not significant.

**Figure 4:**
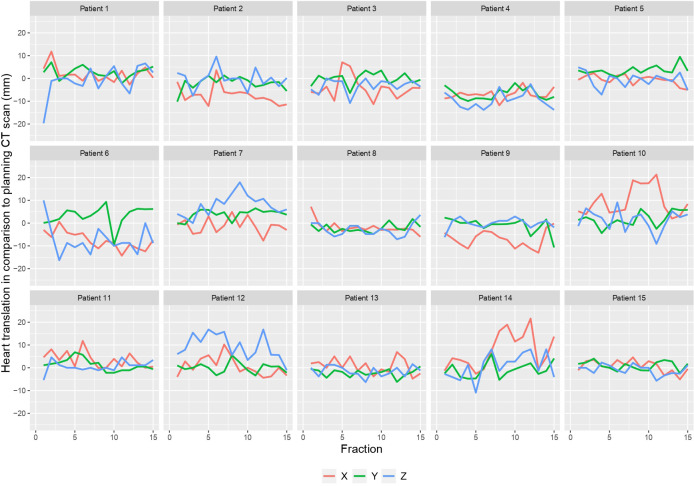
Heart translation per patient in each axis (X, Y, Z) in comparison to the planning CT scan.

**Figure 1** shows the 3D heart motion according to the 225 CBCTs. Linear regression was computed [blue line and the 95% confidence interval (gray area)]. The Spearman correlation coefficients (r_s_) were -0.15 (p=0.025), 0.04 (p=0.549), and 0.03 (p=0.612) for the X, Y, and Z directions, respectively (**Table 1**). The individual 3D heart displacement (in mm) for each patient is presented in **Figure 2**. In total, the median additional translation of the heart relative to the planning-CT during the DIBH course was as follows: X (X_bones_-X_heart_) = 1.1 mm to the right, interquartile range (IQR) = 8; Y (Y_bones_-Y_heart_) = 0.5 mm superiorly, IQR = 4.8; and Z (Z_bones_-Z_heart_) = 0 mm, IQR = 6.4 (**Table 1**, **Figure 3**). The differences in median heart displacement were significant: Friedmann rank-sum test p=0.031, and pairwise comparison using the Wilcoxon rank-sum test was p=0.008 for X and Y; p=0.33 for X and Z; and p=0.07 for Y and Z. **Figure 4** shows these data for each patient on each axis (X, Y, Z). The total median 3D heart motion was δ_tot median_= 7.26 mm, IQR = 6.86 mm.

**Table 1:** Heart translation in each axis (X, Y, Z) and Spearman correlation coefficients.

Axis	X	Y	Z
**Heart translation (mm), IQR**	1.1 IQR = 8	0.5 IQR = 4.8	0 IQR = 6.4
**Direction**	Right	Superior	–
**Spearman correlation** **coefficients**	-0.15 p=0.025	0.04 p=0.549	0.03 p=0.612

mm, millimeters; IQR, interquartile range.

## Discussion

This study investigated the interfraction heart displacement over the course of vDIBH measured by daily CBCT and found intra- and inter-individual heterogeneity (**Figures 1**, **2**, **4**). The displacement was subcentimetric (δ_tot median_= 7.26 mm, IQR = 6.86 mm) and slightly more prominent in the right and posterior directions. Heart radiation dose is a modifiable cardiac risk factor for MCE, highlighting the importance of avoidance of excessive unexpected cardiac exposure.

Recently, analyses on cardiac substructures dose, including the LAD, have been published (19, 20). Zureick et al. found that the dose to the LAD was correlated to adverse cardiac events (19). Additionally, a variation in mean LAD doses was found even among patients with equivalent MHD (19). Interestingly, the mean dose to the atherosclerotic plaque on the “unhealthy” LAD appears to be the best predictor of acute coronary events (20).

Given the cardiovascular risk of breast cancer survivors and the added risk of heart irradiation (21), the heterogeneity of our findings may be crucial in anticipating adverse cardiac events. Nowadays, the potential benefit of DIBH on cardiac morbidity or mortality was only shown by dosimetric parameters and functional imaging (12). Furthermore, monitoring cardiac interfraction motion during vDIBH has never been demonstrated to provide a possible dosimetric benefit. Nevertheless, due to the significant differences in tissue density between the breast, the low-density lungs, and the heart (LAD), unexpected zones of high and low doses may occur (hot and cold spots). The ability to interpret toxicity with the “real daily heart dose” would help to understand the mechanism of heart toxicities and provide guidance on cardiac sparing in RT for treatment and planning.

Our results demonstrated the possible importance of defining cardiac planning organ at risk volumes (PRVs) in an attempt to optimize cardiac sparing and reduce the influence of interfraction motion on plan quality. When determining PTV margins, vDIBH consistency, patient setup reproducibility, and internal motion should all be taken into account (9). In fact, patients could benefit from monitoring the heart position during treatment to avoid unpredictable heart overexposure, especially in the hypofractionated RT situation, where systematic and random errors must be carefully taken into account (22, 23).

Importantly, our findings suggest that patient selection should not be based solely on the initial DIBH planning-CT, and that additional factors should be considered before concluding that a patient is unsuitable for vDIBH. Indeed, during a course of vDIBH, intra- and interfraction organ displacements have been described (12). Rochet et al. and Lee et al. found that parasagittal and axial cardiac contact distances in free-breathing CT scan are correlated to the heart mean dose (24, 25). Based on this, the two UK HeartSpare Studies performed a DIBH planning CT scan only if there was an overlap between the heart and the 50% isodose line on the free-breathing CT scan (13, 14). They excluded 2% of their patients because there was no variation in the heart position between free breathing and DIBH-CT. Furthermore, 2% more were excluded since there was no evident dosimetric advantage (mean heart dose) between the free-breathing and DIBH plans (13). In their study, Kapanen et al. performed vDIBH only if patients could perform a minimum of five shorts (12–15 s) and one long (20 s) reproducible (2 mm) vDBIH (26).

vDIBH is a method that may potentially decrease anxiety, while also possibly improving treatment compliance and experience. The current method’s strengths include its emphasis on daily patient preparation and motivation, as well as its avoidance of vDIBH and IGRT repetition and replanning, which might result in time and resource saving. According to Kapanen et al., a high number of apneas required by vDIBH may increase superior–inferior (SI) intrafractional displacement while diminishing vDIBH efficiency due to the patient’s exhaustion and stress (26). Since the heart motion in this direction (SI) was minimal (0.15 mm) in our study, vDIBH without visual feedback seems to be effective in reducing variability in this SI axis (**Figure 3**).

Unfortunately, most of the literature focusing on DIBH reproducibility and stability focused on a single DIBH session rather than the full course of RT (26–30). It is well known that the breast tissue could vary during RT (e.g., seroma, edema, shrinkage) and move according to respiration (31, 32). Gierga et al. suggested that a variation between internal and external anatomy could happen during treatment (33). Nonetheless, Reitz et al. found an intrafractional deviation (<5 mm) during DIBH using a surface-guided technique. In contrast to our 3D assessment, their study only examined the vertical deviations to determine DIBH stability and reproducibility (33).

The risk–benefit of breast RT varies between studies and may not be favorable for all women equally (e.g., long-term smokers, techniques, fields) (2, 4). Furthermore, some physiological breathing variation (chest vs. abdomen) could still occur (9, 34). Indeed, in contrast to cardiac motion, which is rhythmic, respiratory motion is “involuntary” and nonrhythmic (9). Throughout imaging and treatment sessions, patients’ breathing patterns can change in magnitude, frequency, and consistency (9). In order to sustain a consistent vDIBH, the patient should be given adequate rest before performing the next vDIBH.

**Figure 5:**
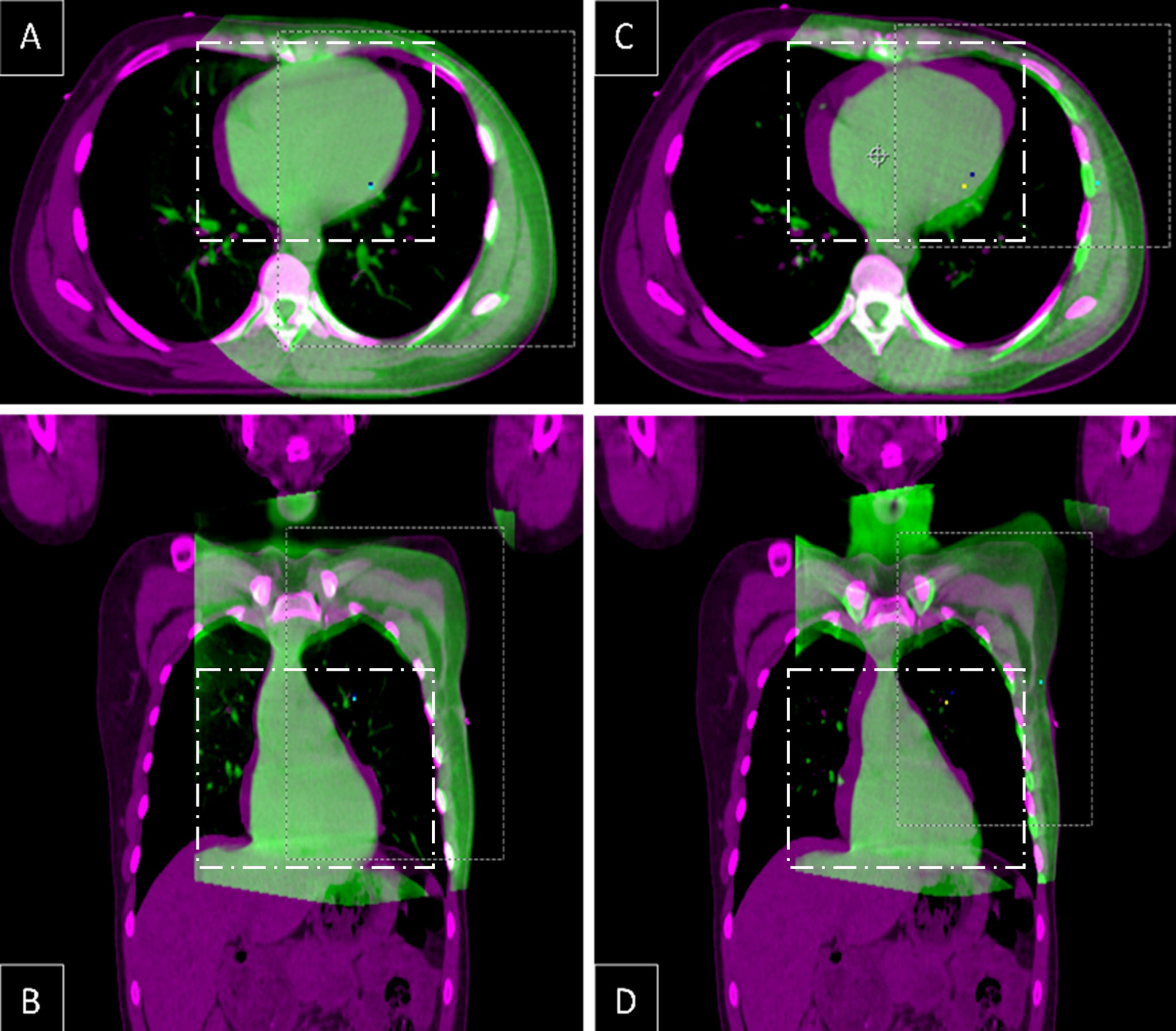
Fusion of axial **(A, C)** and coronal **(B, D)** CT slices from the same level in DIBH planning CT (pink) and the first **(A, B)** and the last **(C, D)** CBCT (green), showing interfractional heart translation.

In this context, the role of RTT, who should provide standardized coaching and support to patients, is crucial. In the present study, we assessed all the fractions and performed a 3D CBCT matching (**Figure 5**). We did not find any clinically meaningful improvement in the heart position over a course of vDIBH.

vDIBH is a challenging task, and practitioners must be aware of the wide range of intra- and inter-individual heart position variation. Patient selection may not be solely based on the DIBH planning-CT. The use of a combination of audible and visual input may enhance homogeneity, but each patient must be evaluated personally (9). DIBH coaching and home practice (at least five days) could significantly decrease the cardiac dose (16).

Our findings must be considered in light of potential limitations. The heart displacement was measured using the CBCT timepoint, and the intrafractional heart movement was not assessed. Moreover, during vDIBH, the heart beating motion is present and could create an artifact during the CBCT acquisition. Secondly, we assessed the displacement of the whole heart without considering the different parts of it (e.g., LAD). Finally, because no visual feedback was provided during CBCT, different results may be obtained if patients get both audio and visual feedback in order to perform vDIBH in the same way as the CT simulation. However, this also presents advantages given the patient is free to perform a vDIBH as they want every day. While our methodology and data indicate innovation and potential, a larger investigation is required to strengthen results even more.

## Conclusion

Breast radiotherapy emphasizes consistency and accuracy when it comes to the localization of vital organs such as the heart. Clinicians must be conscious of the range of intra- and inter-individual heart position variation during DIBH even if SGRT is used. The DIBH planning-CT may not be sufficient to select candidates. Patients may benefit from heart position monitoring during therapy to minimize unplanned cardiac overexposure. The inter-individual variation found in our study could be taken into account for developing more accurate heart dose-volume models.

## Data availability statement

The original contributions presented in the study are included in the article/supplementary material. Further inquiries can be directed to the corresponding author.

## Author contributions

Study design: SB and ADC. Data collection: SB and ADC. Data analysis and interpretation: SB and ADC. Writing of the manuscript: SB, CGdS, YJ, and ADC. Revision of the manuscript: All authors. Statistical analysis: SB and ADC. All authors contributed to the article and approved the submitted version.

## Conflict of interest

The authors declare that the research was conducted in the absence of any commercial or financial relationships that could be construed as a potential conflict of interest.

## Publisher’s note

All claims expressed in this article are solely those of the authors and do not necessarily represent those of their affiliated organizations, or those of the publisher, the editors and the reviewers. Any product that may be evaluated in this article, or claim that may be made by its manufacturer, is not guaranteed or endorsed by the publisher.
